# Phlebotomy in Practice: Evaluating the Impact of Timetabled Clinics on Medical Student Venepuncture Skills

**DOI:** 10.7759/cureus.107400

**Published:** 2026-04-20

**Authors:** Kristen Medalla, Win Mon, Anila Viepadan

**Affiliations:** 1 Medical Education, Great Western Hospitals NHS Foundation Trust, Swindon, GBR; 2 General Practice, Royal United Hospital, Bath, GBR; 3 Medical Education, Dartford and Gravesham NHS Trust, London, GBR

**Keywords:** clinical simulation, clinical skill teaching, confidence level, educational supervision, learning resources, phlebotomy, venepuncture, venipuncture

## Abstract

Background

Venepuncture is included among the list of clinical skill competencies in the current General Medical Council (GMC) outcomes for medical graduates. Traditionally, medical students learn venepuncture skills in simulation first, followed by practice in clinical environments. However, clinical practice depends largely on available opportunities such as ward and clinic time.

Aim

This study aims to identify the impact of a scheduled phlebotomy clinic on the confidence levels of medical students in performing venepuncture and the reduction in the level of future supervision needed to perform the skill on the wards.

Methods

A questionnaire was sent to 41 medical students, 11 of whom attended the phlebotomy clinic. The questionnaire consisted of Likert scales, multiple-choice questions and free-text questions to assess their confidence levels in venepuncture. The Mann-Whitney U test was used for quantitative statistical analysis, and thematic analysis was implemented to assess qualitative data.

Results

Students' confidence in performing venepuncture increased after attending the phlebotomy clinic (95% CI: 0.672 to 0.973, p<0.0005). Confidence in performing venepuncture unsupervised was higher in those who attended the clinic, compared to those who did not attend (95% CI: 0.165 to 0.509, p<0.0292). The key theme that emerged from the comparison group was that students wanted more opportunities to practise venepuncture skills on real patients.

Conclusion

Our study shows that having the opportunity to attend a phlebotomy clinic can positively impact the self-reported confidence levels of students when performing venepuncture. The students expressed that they would like more opportunities to practise venepuncture in the clinical environment, especially on patients and whilst supervised. Therefore, implementing regular phlebotomy clinics in their placement could be a huge benefit to medical students.

## Introduction

The General Medical Council highlights a list of clinical skill competencies which are required prior to graduation [1], one of which is venepuncture. Performing venepuncture requires the healthcare professional to be competent in both clinical knowledge and dexterity, built through practice [2]. Venepuncture is most often learned in a stepwise fashion - students are first assessed and signed off as competent in a simulated environment prior to performing in the clinical environment. Supervision is reduced with practice, as student confidence and experience increase [3]. There is, however, little evidence in the literature suggesting that once the skill is signed off in a simulated environment, medical students will be competent and confident to practise in the hospital wards under supervision. Furthermore, there has been a varying degree of correlation between self-assessed confidence of the learner and an assessment of the clinical skills conducted by the educator [4].

The quality and depth of both theoretical and practical teaching can vary significantly between teaching hospitals [5]. Several factors can limit the effectiveness of this teaching, including clinician availability and resource constraints, and can form a barrier in maximising the number of venepuncture attempts available during a session [6]. Simulation tools, such as phlebotomy mannequin arms, are often pre-prepared and fail to replicate the experience of locating real veins [7]. Overall, the simulated environment lacks many of the challenges and unpredictability of the real-life clinical setting. It is, therefore, important to recognise the need to bridge the learning gap between mastery of venepuncture in a simulated environment and a clinical environment. This study aims to assess the impact that one timetabled phlebotomy clinic has on medical students' confidence in performing venepuncture and to reduce the level of future supervision needed to perform the skill on the wards.

## Materials and methods

Study design

This study was conducted at Great Western Hospital, a UK teaching hospital, and all students recruited attended placement in this hospital. This was a prospective, observational study conducted over a five-month period in 2025. A phlebotomy clinic was timetabled for each of the 11 students on the same weekday morning clinic on consecutive weeks (January 2025-May 2025) before the six-week data collection period. The confidence levels of these medical students were assessed following the introduction of this phlebotomy clinic and then compared to the typical medical student experience in venepuncture practice.

The primary outcome of the timetabled phlebotomy clinic was to assess changes in the self-reported confidence. The secondary outcomes included assessment of qualitative perceptions, especially in factors improving medical student experience in learning venepuncture.

Inclusion and exclusion criteria

The intervention group consisted of 11 students from the same university cohort, who were timetabled for a half-day phlebotomy clinic during their placement. Prior to the clinic, these students attended a clinical skills session covering venepuncture techniques in a simulated environment, incorporating key anatomical and procedural theory, trust policies and learning resources provided to phlebotomy trainees. The clinical skills session provided a practical opportunity for students to practice venepuncture on mannequins under the supervision of a trained clinician, and sign off as competent to practise on the wards. No set number of failed attempts was assessed during their formative assessment in the clinical skills session; however, students are required to pass this prior to practising in the clinical environment. During the clinic, they were able to shadow a phlebotomist as well as have further supervised practice of venepuncture with real patients.

The comparison group consisted of year 3 to year 6 medical students from three different universities, who were on placement on site at the hospital at the time of the study. Similar to the intervention group, the comparison group also had prior experience of formally being taught venepuncture in a simulated environment, and were recruited through word-of-mouth in teaching sessions, posters and email invites. The comparison group represented the typical medical student experience of learning venepuncture, and these students did not have a scheduled phlebotomy clinic in their rotation at the time of data collection. No specific exclusion criteria were implemented, as all students recruited were on clinical placements and had been formally taught venepuncture in clinical skills sessions prior to the survey being sent.

Data collection

A questionnaire was sent out to all participants, as demonstrated in Appendices A-C. A period of six weeks was allocated for data collection, resulting in all 11 students from the intervention group and 30 students from the comparison group completing the survey. Appendix D shows how the data were selected from both groups. Qualitative and quantitative data were retrieved to assess their confidence levels, current experiences and teaching quality related to venepuncture.

The intervention group was also evaluated for changes in confidence after the clinic. Informed consent was obtained on the online form with details of the study, prior to completing the survey. Data has been stored securely in compliance with data protection regulations, and findings were anonymised before publication.

Statistical analysis

A formal sample size calculation was not performed, as a purposive sample was recruited based on all eligible students (n=104) available within the defined study period.

The questionnaires sent out consisted of both qualitative and quantitative questions. For the qualitative data, open-spaced questions were utilised. For the quantitative data, a mix of Likert scales and multiple-choice options was used. The questions were created after an extensive literature review of relevant research papers, and a comparison of previous questionnaires was implemented [2,4,8-11]. The questionnaire then underwent peer-to-peer review by different clinical teaching fellows who have had experience in research as well as teaching medical students. The qualitative data were analysed, and similar themes were identified within each question, using Braun and Clarke’s six phases of thematic analysis framework [12]. The Mann-Whitney U test was used to assess the difference in student confidence levels in performing venepuncture supervised and unsupervised, and whether there is a change following the introduction of a phlebotomy clinic.

## Results

Learning modalities and resources for venepuncture skill

**Figure 1:**
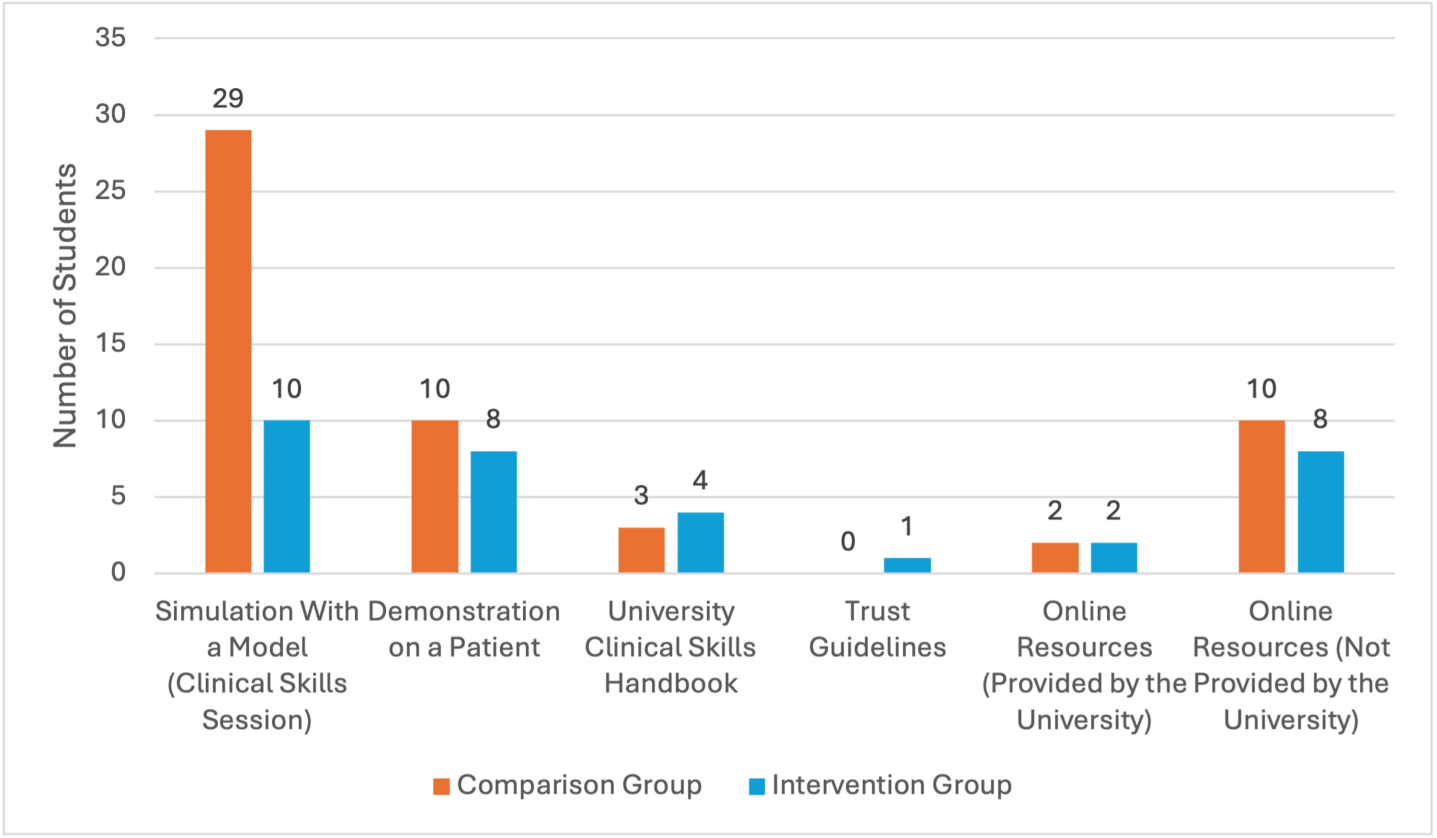
Learning Modalities and Resources Utilised by Students for Venepuncture Skill

A range of learning resources was used to introduce venepuncture skills and knowledge, as seen in Figure 1. The most common modality was "Simulation with a Model/Clinical Skills Session" for both the comparison and intervention groups (n=39, 95.12%), followed by "Demonstration on a Patient" and "Online Resources Not Provided by the University" (both n=18, 43.9%).

While most students in the intervention group reported that formal teaching provided adequate preparation for real-life venepuncture (n=10, 90.90%), there was a different opinion amongst the comparison group (n=12, 40.00%). Some students in the comparison group (n=13, 43.33%) stated that formal teaching did not provide adequate preparation, and a minority of students (n=5, 16.66%) were unsure. Using Braun and Clarke’s six phases of thematic analysis framework [12], further evaluation into student perception of the formal teaching was consistent in both groups: mainly that the teaching provided opportunities to practice the correct technique on a model and receive feedback in a safe environment. Feedback on potential improvements included having more sessions in general, giving specific advice on how to identify veins on a patient compared to the mannequin, having more opportunities to perform venepuncture on patients, additional supervision on the wards, and attendance at phlebotomy clinics. Both groups identified the limitations of simulated teaching, reflecting the complexity of real-life scenarios.

Venepuncture in the clinical environment

**Figure 2:**
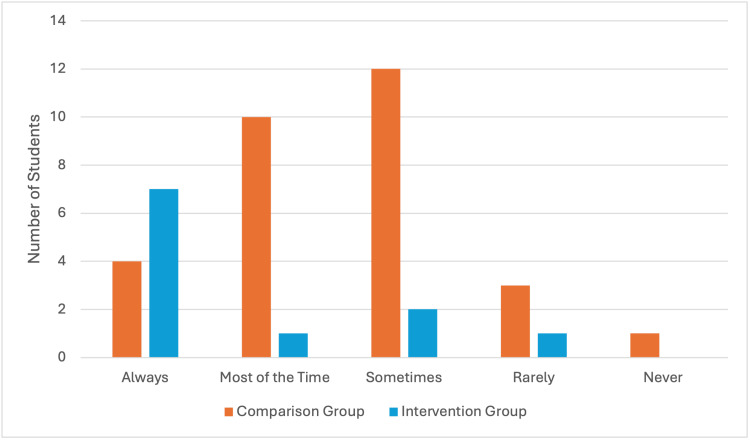
Supervision by a Healthcare Professional When Performing Venepuncture on the Wards

Both the comparison and intervention groups showed higher confidence levels in venepuncture when supervised, with an average of 8.36 and 7.5, respectively. However, both groups showed variable experience in supervision when practising on the wards, as demonstrated in Figure 2. This average self-reported confidence was significantly higher than when unsupervised, with scores of 5.7 in the comparison group and 7.09 in the intervention group.

Similar themes were identified in both groups on factors which helped students practise venepuncture on the wards, including supportive staff who provided opportunities to practise, as well as students themselves being proactive to find time and people to help them.

**Figure 3:**
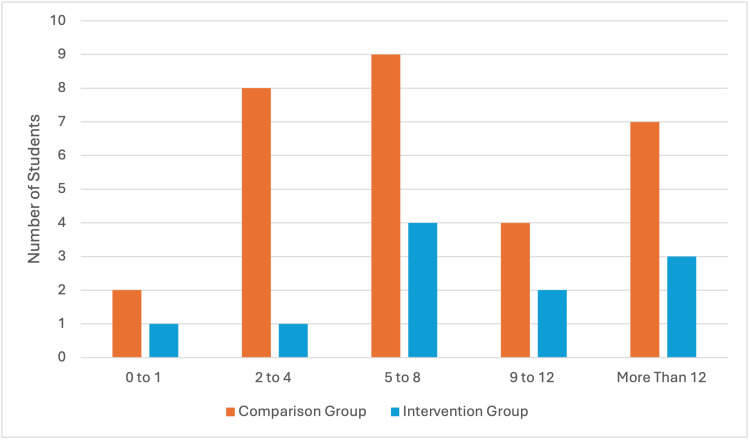
Number of Venepuncture Practice on Placement Amongst Students Both Supervised and Unsupervised

Students reported that some of the biggest challenges in performing venepuncture on real patients included identifying adequate veins, patients not consenting to students carrying out the procedure, anxiety about their ability and causing pain to patients. Other themes identified within the comparison group included encountering unfriendly staff members, lack of supervision and reduced opportunities to practise in the first place, as seen in Figure 3. Student experience on receiving adequate feedback and guidance is mixed, with a higher proportion of students in the intervention group reporting that they received sufficient guidance and feedback when practising venepuncture on the wards (n=9, 81.81%), as opposed to the comparison group (n=12, 40%).

Confidence level in the intervention group post-phlebotomy clinic

There was a significant increase in average self-reported confidence levels from 5.55 to 8.18 following the introduction of the phlebotomy clinic. All students reported that attending the phlebotomy clinic improved their venepuncture skills and that the balance between formal teaching, ward practice and additional training was sufficient for learning venepuncture. Students found the phlebotomy clinic particularly useful when there was a phlebotomist demonstration and guidance (n=2, 18.18%), being able to practise on real patients (n=1, 9.09%), repetition and multiple opportunities for practice (n=5, 45.45%) and completing the paperwork in a clinical setting (n=1, 9.09%). Suggestions to improve the clinic included providing the same needle type used in the clinic during clinical skills sessions, increasing the number of clinic sessions and offering opportunities to shadow a phlebotomist on the wards.

## Discussion

Learning in the clinical environment

The results demonstrated that students engaged in proactive practice of venepuncture skills, with most students receiving supervision from healthcare professionals. Appendices E-I show the identified limitations, which included staff being unable to balance supervision with clinical responsibilities, a lack of patient consent and identification of adequate veins, and maintaining patient safety. Despite these barriers to learning, the majority of students reported that they were able to receive adequate learning opportunities. Students from both groups expressed lower confidence levels when performing venepuncture on patients unsupervised, indicating the importance of having regular supervision during teaching sessions. Studies have shown that deliberate practice of clinical skills under the supervision of an engaged instructor is a key component of the mastery model [13] and that merely performing skills without adequate feedback has no effect on long-term competence [8]. Therefore, we can assume that feedback, combined with the hands-on learning experience, is fundamental in not only ensuring technique refinement but also increasing student confidence.

Introduction of scheduled phlebotomy clinics

The results clearly showed that students’ self-reported confidence levels in performing venepuncture increased after attending the phlebotomy clinic (95% CI: 0.672 to 0.973, p<0.0005). Moreover, the confidence level within the phlebotomy clinic group was higher in performing the skill unsupervised (median difference 1, 95% CI: 0.165 to 0.509, p<0.0292) than the comparison group. Themes identified within the control group regarding improvement in confidence were interrelated. For example, dedicating teaching time in clinics and wards to perform venepuncture enabled both additional practice and provided patient-based teaching opportunities. This is supported in research suggesting that higher fidelity simulations not only improve knowledge and memory retention, but also self-confidence in applying skills in more challenging environments [14]. Confidence levels among students performing the skills when supervised, however, were not statistically significant (95% CI: 0.213 to 0.572, p<0.1029), but this outcome could be attributed to the unequal sample sizes of the intervention and control groups.

It is also important to consider that the confidence in performing clinical skills builds on basic procedures to more complex ones. Performing venepuncture requires several steps - preparation, obtaining consent, carrying out the procedure and tidying up - similar to other clinical procedures. A study showed that participants who felt confident in performing phlebotomy skills without supervision were also more likely to perform other skills, such as arterial blood gas sampling and nasogastric tube insertion [9]. Therefore, building student confidence in performing venepuncture unsupervised from the early stages of training is crucial in developing more advanced clinical skills.

A limiting factor to consider in implementing scheduled phlebotomy clinics in medical student teaching is balancing additional key skills, knowledge and experiences in a structured curriculum. This study could also be subject to a potential risk of self-selection bias as recruitment of students was voluntary, therefore limiting the generalisability of the findings to the wider student population. Moreover, the impact of this modality of teaching can vary depending on the year group of medical students, particularly when considering the task complexity in the application of real-life clinical practice [15]. While this teaching strategy could maximise the opportunities for students to practise their venepuncture skills, patient safety needs to be considered, for which having a healthcare professional supervising them in the earlier stages might be beneficial. As medical students develop their clinical skills and experience, a phased approach to learning, through gradually reduced supervision, is recommended. An example of such a phased pedagogical strategy for procedural skills is the "Learn, See, Practice, Prove, Do, and Maintain" framework through which the learner develops competency in procedural skills with increasing experience [16].

Learning resources

Supplementary learning resources have also been a key factor in clinical skill development. A significant number of students in the intervention group have reported that the use of external resources, rather than university-provided materials, assisted their venepuncture training. This finding highlights the potential gaps in the current formal teaching provided by the university and provides scope for developing these resources as a revision tool.

There is increasing research into identifying strategies to teach venepuncture skills, with one literature highlighting that the best patient outcomes were achieved when students received theoretical content, such as lectures, e-learning and demonstrations, in an educational setting first, then on a mannequin after [17]. This is well-supported in a study by Lindenmaier et al. [2], which suggests that e-learning can increase student academic competence when used alongside traditional learning. Similarly, these laboratory-based trainings can also develop professionalism and build on similar transferable skills, such as cannulation, before working in the clinical environment [18].

Within our study, medical students were given access to the learning resources developed for phlebotomy students; therefore, this study identifies further research opportunities into how learning resources from allied healthcare professionals can be applied in clinical skills that overlap in medical training. However, there are notable differences in the training requirements and competency expectations among other health professionals who perform similar clinical skills. For example, nursing competency is dictated by a set number, often greater than the medical student requirement, of supervised and unsupervised procedures that must be completed [10]. Therefore, further evaluation is warranted to determine whether the medical curriculum should structure clinical skill teaching around a specific number of completed or successful attempts.

Assessment of competence

Human factors play a role in the quality of the teaching the students receive on the ward, as well as student confidence. The qualitative data shows how anxiety, combined with negative staff behaviour, impacts their ability to learn. Although all students were signed off as competent to perform the skill in a simulated environment, they reported anxiety about their ability to perform the skill competently in the clinical environment. Other key determinants associated with clinician confidence include gender, total number of procedures performed, and number of procedures performed in the previous year [11,19].

The disparity between the student being signed off as ‘competent’ with their confidence and experience on the wards suggests that these factors should be incorporated into the assessment of clinical skill competence. The outcomes of this study are based on self-reported confidence, which may not directly correlate with objective clinical competence. In fact, Barnsley et al. have demonstrated a lack of relationship between self-reported confidence and competence in typical clinical skills expected from a newly qualified doctor, with many inadequacies ranging from applying sterile or aseptic technique, documentation and familiarity with the equipment [11]. A potential way to improve this could be to simply allow for more opportunities for students to build confidence and develop their skills in the clinical environment [20], particularly in procedures they will typically see early in their career [11,21].

One of the limitations of the study is the variability of prior learning experience of venepuncture skills. This individual difference could result in a disparity between student confidence and competency level pre- and post-intervention. Further research would be recommended with a more standardised level of prior experience to study whether other strategies, such as Direct Observation of Procedural Skills (DOPS), should be applied in assessing venepuncture competency [22]. Due to a technical error, two students were unable to complete Questions 14 and 15 on the survey, and, therefore, the results from these questions were unable to represent all the responses from the comparison group. Analysis of the data was based on available responses only.

## Conclusions

Our study shows that having the opportunity to attend a phlebotomy clinic can positively impact the self-reported confidence levels of students when performing venepuncture skills. From the qualitative data we received, a core theme that emerged, regardless of the students’ prior experience, was an increased opportunity to practise their venepuncture skills in the clinical environment. The students also valued practising their venepuncture skills on patients in a supervised environment so that they can receive feedback.

Other themes that we obtained included providing further learning resources to students during their formal theory teaching, as well as addressing any anxiety that students may feel when performing venepuncture. Therefore, implementing timetabled phlebotomy clinics as part of medical students’ scheduled learning during their clinical placement could be beneficial, as it would provide more opportunities and supervision, all of which can aid in increasing confidence.

## Figures and Tables

**Figure 4:**
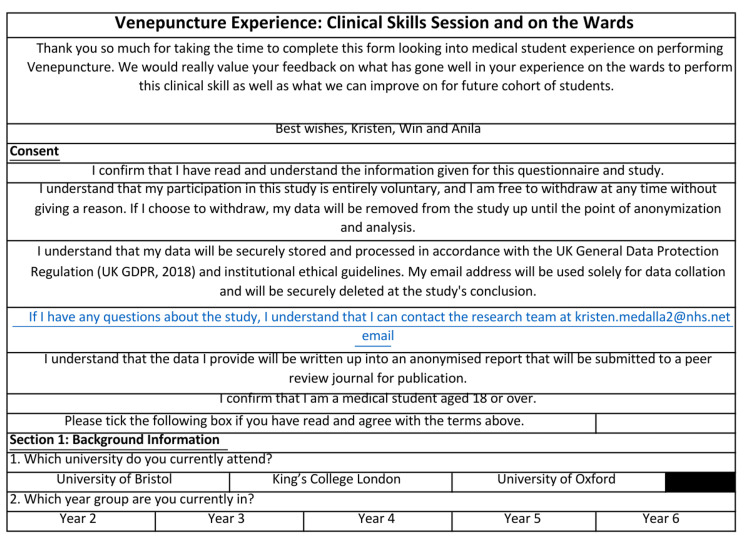
Medical Student Feedback Form - Consent and Background Information

**Figure 5:**
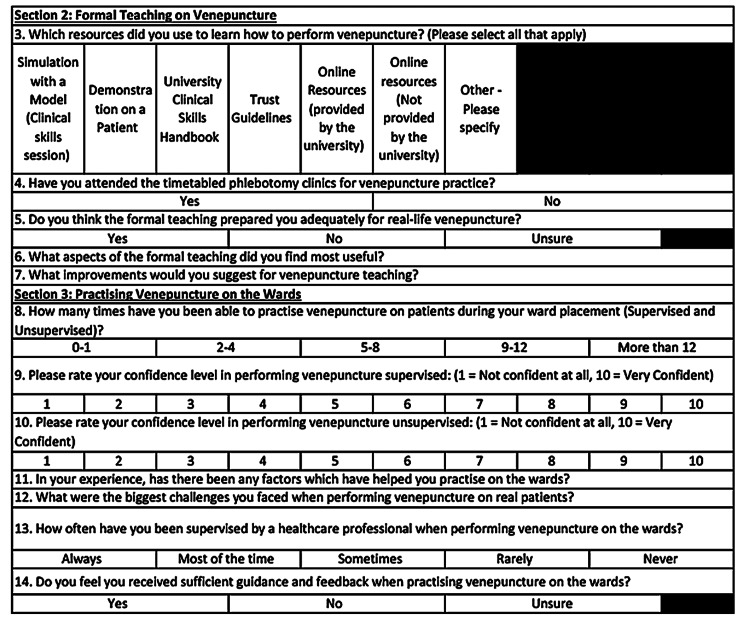
Medical Student Feedback Form - Formal Teaching and Ward Experience

**Figure 6:**
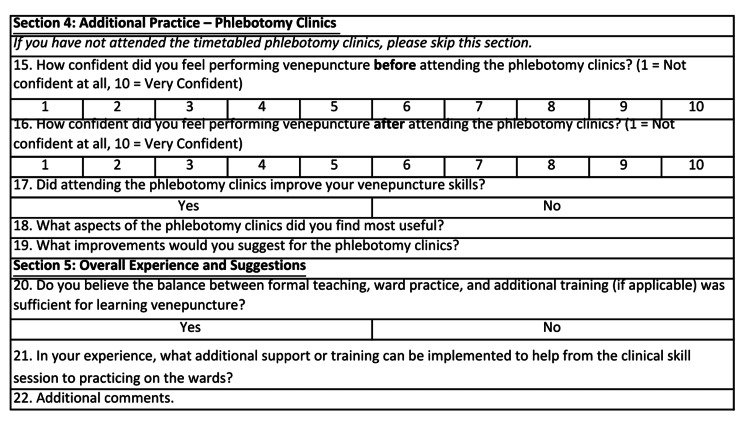
Medical Student Feedback Form - Phlebotomy Clinics and Suggestions

**Figure 7:**
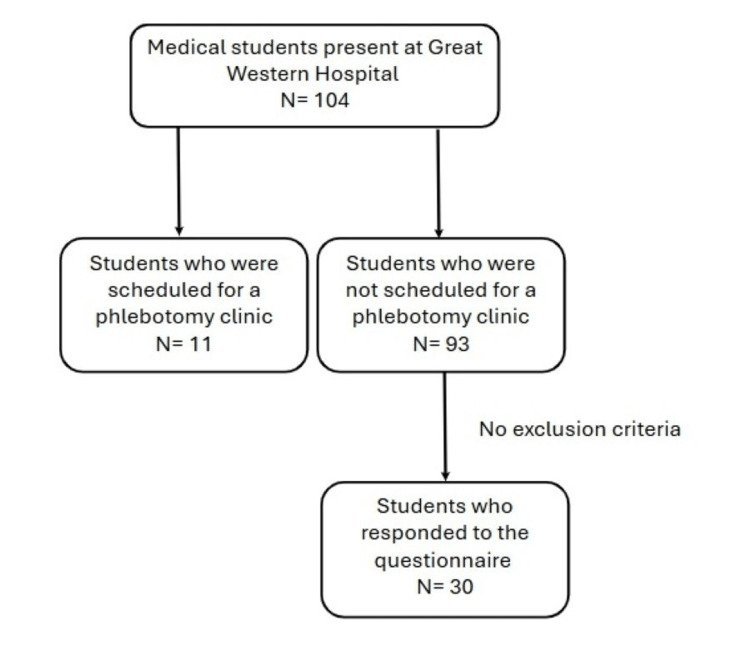
Student Selection Process

## Appendices

Appendix A

Medical Student Feedback Form

Appendix B 

Appendix C 

Appendix D

Cohort Flow Chart for the Student Selection Process

Appendix E

Thematic Analysis Tables for Qualitative Data From Both Comparison and Intervention Groups

**Table 1:** Thematic Analysis of Question 6 on the Medical Student Feedback Form - "What Aspects of the Formal Teaching Did You Find Most Useful?"

Themes	Quotes
Practice	"Practising on model before real patient." "Practising on mannequins."
Demonstration	"Demonstration on how to perform venepuncture on a mannequin - actually seeing it in action." "Demonstration on a patient."
Opportunity	"Having multiple opportunities to practice."
Tutorials	"Tutorial on procedure." "Understanding the process of how to take blood."
Guidance	"Someone talking through the technique." "Tips and tricks on how to handle difficult veins."
Psychological Safety	"Being in a safe environment." "Being able to make mistakes."

Appendix F 

**Table 2:** Thematic Analysis of Question 7 on the Medical Student Feedback Form - “What Improvements Would You Suggest for Venepuncture Teaching?”

Themes	Quotes
More time	“More practice made available.” “More time to practice I feel like they always are very rushed.” “More sessions.”
Teaching in clinics	“Phlebotomy clinics would be really helpful.” “Phleb clinic as part of placement.” “Time in phlebotomy clinics.”
Teaching on ward	“Would be good to have teaching on the ward.” “Allowing scheduled time to go to acute assessment units to practice.”
Teaching on patients	“More time to practice on patients.” “More supervised practice on patients.” “Actually having time to practice on real people.”
More shadowing	“More opportunities to observe real life demonstration.” “More time under supervision.”

Appendix G 

**Table 3:** Thematic Analysis of Question 11 on the Medical Student Feedback Form - "In Your Experience, Has There Been Any Factors Which Have Helped You Practice on the Wards?"

Themes	Quotes
Friendly staff	“Having a nice clinician to approach.” “Nice friendly staff.” “FY1s to give me encouragement and confidence.”
Being proactive	“Going to ED and talking to the HCAs who were really keen on teaching.” “Be proactive and engaging.” “Asking doctors who needs bloods.”
Having opportunities	“More opportunities.” “Having scheduled time.”
Supervision	“Having the fy1 being able to help me makes me feel much more confident.” “When doctors take the time to watch and support you.” “Practising supervised with all the equipment ready.”

Appendix H 

**Table 4:** Thematic Analysis of Question 12 on the Medical Student Feedback Form - "What Were the Biggest Challenges You Faced When Performing Venepuncture on Real Patients?"

Themes	Quotes
Anxiety about ability	“Fear of failing or getting shouted.” “Getting flustered.” “Worried you'll do it wrong.”
Difficult access	“They didn’t have any accessible veins.” “Having more frail patients who don’t have obvious veins.”
Anxiety about causing pain	“Don’t want to hurt them.” “Thinking about the patients being in pain.”
No opportunity	“Opportunity to actually take bloods from patients.” “Finding the opportunity to do it.”
Unfriendly staff	“On the wards staff weren’t nice.” “Fear of ... getting shouted at by staff.”
No supervision	“Not having supervision.” “Not being able to be supervised.”

Appendix I 

**Table 5:** Thematic Analysis of Question 21 on the Medical Student Feedback Form - "In Your Experience, What Additional Support or Training Can Be Implemented to Help From the Clinical Skill Sessions to Practising on the Wards?"

Themes	Quotes
Phlebotomy clinic	“Phlebotomy clinic.” “Phlebotomy clinics where a student who run the clinic.” “More time with phlebotomists.”
Practice on real patients	“More on real people.” “Need more opportunities to practice with real people.”
Supervision	“Dedicated supervised practice.” “Practise under supervision with feedback.”
More teaching	“Maybe include it into bedside Teaching.” “More teaching on real life patients.” “More practise sessions.”
